# Primary Health Care Center (PHCC) Location-Allocation with Multi-Objective Modelling: A Case Study in Idleb, Syria

**DOI:** 10.3390/ijerph16050811

**Published:** 2019-03-06

**Authors:** Pınar Miç, Melik Koyuncu, Jamil Hallak

**Affiliations:** Department of Industrial Engineering, Çukurova University, Sarıçam 01330, Turkey; mkoyuncu@cu.edu.tr (M.K.); jhallak@student.cu.edu.tr (J.H.)

**Keywords:** Syria, primary health care center (PHCC), location-allocation, multi-objective model, weighted goal programming, geographic information system (GIS), analytic hierarchy process (AHP)

## Abstract

The Syrian crisis began on 15 March 2011. It is one of the bloodiest and complicated conflicts in the world today. Although almost eight years have passed over this tragedy, civilians continue to suffer from conflicts and destructions in the area. As a result, this situation disregards human life and the number of people in need increases day by day. Particularly, people who have to live in the conflict area encounter troubles with regard to health, shelter, food and other needs. Thus, we have focused on identifying the Primary Health Care Center (PHCC) locations within Idleb Governorate in Syria. Data is extracted from a sample containing 23 sub-districts in the governorate and a total of 338 communities. We have formulated a mixed integer-weighted goal programming model and combined it with a Geographic Information System-GIS (ArcMap). The model is solved via an optimization package and moreover, sensitivity analyses are conducted to achieve a more in-depth study. Our aim was to have 60 PHCCs out of 77 available candidate PHCCs and the model located 42 PHCCs in total, by allocating 379,080 people, with a total cost of USD 1,000,353 and a cash for work amounting to USD 163,549. Accordingly, the model’s outputs and sensitivity analyses are expected to help decision-makers in case of such disasters.

## 1. Introduction

Disasters may occur anywhere in the world and may be categorized as natural or man-made disasters. While a natural disaster emerges from natural hazards on earth (such as floods, earthquakes, volcanic eruptions, hurricanes, tsunamis, etc.); man-made disasters are caused by human behaviors. Some of these include, among others, industrial, transport or public health accidents, terrorism, crimes against humanity and warfare. It is crucial to state that warfare may be one of the most devastating types of man-made disasters as it has both severe and long-term implications. Even though the warfare or conflict ends, serious problems such as its damages to the country, environment, infrastructure, healthcare services; the lack of food, water and other resources, displaced people and disease outbreaks may continue to be prevalent [1]. 

Warfare and other conflicts have materialized many times across the globe. Wars have further damages in addition to the killing of people. Among these damages we can count forced migration and displacement as important ones. Nowadays, The Office of the United Nations High Commissioner for Refugees (UNHCR) estimates that there are approximately 68.5 million people who are subjected to forced displacement at global level worldwide and have been forced to leave their homes because of battles and armed conflicts [2]. Of these 68.5 million people, about 40 million are internally displaced people (IDPs); approximately 25.4 million are refugees and nearly 3 million are asylum seekers [2]. While a refugee is defined as someone who has been forced to run away from his/her own country due to persecution, war or violence, IDPs are described as the people or group of people who have left behind their home, habitual residence and livelihood, but have not crossed any international border [2]. Even though their own country is the source of their displacement, IDPs proceed to wait for protection by their government. The harsh fact about refugees is that the citizens of only three countries constitute more than half of 24.5 million refugees worldwide: South Sudan (about 2.4 million), Afghanistan (nearly 2.6 million) and the Syrian Arab Republic (approximately 6.3 million). This demonstrates that the Syrian Arab Republic has the highest share among these three countries and it has led us to the Syrian Crisis which began in March 2011. It became an international crisis from that day on and accounted for the largest migration of people since World War II. It materialized as a crucial humanitarian emergency through the establishment of refugee camps, seeking for shelter and the presence of thousands of asylum seekers in different countries. These humanitarian emergencies devastate the local government’s capacity to handle and provide affected populations with basic necessities, such as food, clothing, shelter, water, non-food items (blankets, sheets, cooking items, soaps, etc.), security and healthcare facilities. Thus, refugees’ and IDPs’ safety and healthcare needs have evolved into a more and more significant topic all over the world. The Syrian Crisis is a typical example of health challenges encountered by refugees, IDPs and host countries [3]. Due to troublesome conditions under which they live, they are defenseless and various factors including fatigue, the lack of food and clean drinking water as well as poor hygiene influence their health [4]. 

Hence, the planning of healthcare response to natural and man-made disasters has attracted more attention in the last decades [5,6,7,8,9]. In this respect, it is noteworthy to point out primary healthcare centers (PHCCs) at local level as they are very vital to offer health facilities and services. During a disaster, irrespective of being natural or man-made, PHCCs play a critical role in saving as many lives as possible. They constitute the key structural and operational part of public health services in developing countries, as in Syria. It is difficult to estimate requirements and figure out the allocation of PHCCs and appropriate resources, particularly in the case of any humanitarian emergency like battles and forced migration such as the Syrian case and accordingly, this situation poses an enormous danger. Although forced migration in the area is a long-lasting process and has apparent threats to the health of the population, it is not exactly categorized as a global health issue, yet [10]. On the contrary, responses to the health needs stemming from the excess number of forcibly-displaced people have proven to be insufficient to a large extent.

To gain a better understanding of the subject, we have reviewed the related literature under two titles: “primary health care centers in disasters” and “location-allocation problems in disasters.”

Before researching literature for primary health care centers in disasters, we reviewed health issues in disasters. For the issue of health within natural disaster settings, studies were rare and we realized that a great majority of studies were based on earthquakes [11,12,13,14,15]. For the issue of health during man-made conflict settings, the studies can be collected under five titles: disaster response [16], health system performance [17,18], health activities and capabilities [19,20,21,22]; psychosocial issues [23,24,25,26] and health requirements [27,28]. 

In respect to PHCCs within natural disaster settings, some studies focused on earthquakes [29], hurricanes [30] and floods [31]. Other studies included: a national study of connections among health centers and the emergency preparation and response plan attempted in their own societies [32], analyzing the level of disaster preparedness in public hospitals and applying the hazard vulnerability analysis (HVA) tool [33], developing and accessing health care in disrupted societies and studying frameworks in primary care [34], and evaluating health-care providers’ insights of their knowledge, abilities and preparedness for disaster management [35]. 

In relation to PHCCs in armed conflict settings, some studies focused on: access to water and sanitation [36], non-governmental organizations’ (NGOs) role in delivering therapeutic health services in conflict settings [37], the integration of staff’s well-being into the primary health care (PHC) system [38], non-communicable diseases (NCDs) among refugees [39] and the PHC’s role to promote an incorporated distribution of care to refugees [40]. Furthermore, some studies focused on the scope of mental, neurological and substance use (MNS) services in refugee camps [41]; specifying the problems and summarizing the precedence’s and obstacles in negatively influenced health care systems by armed conflict [42]; medical condition, unfulfilled requirements and the provision of health services among refugees [43] and health workers’ multifactorial behavioral interference to the adults in the primary care settings regarding conflicts [44]. In conclusion, many researchers have studied primary health care subjects in natural and man-made disasters, but there is no literature related to the location-allocation problem regarding primary health care centers.

With regard to location-allocation problems in medical settings during or in preparation for disasters, which is also the focus of this study, some studies adopted multi objective models for the purposes of covering as many patients as possible [45] and of minimizing: the travel time of patients [45,46], queue duration [45], the total mortality risk value of patients [46], total response time by taking costs into consideration [47,48], total network access time and the operating costs of a shelter and the cost of failure to accommodate all evacuees [49], the total demand-weighted transportation time between facilities and the cost of lost demand at the points of demand and hospitals [50], relief distribution network [51], expected costs over all scenarios [52] and the total sum of distance plus expected value [53]. 

Since the Syrian war is currently the most complicated and bloodiest conflicts in the world, the humanitarian community is expected to provide emergency and life-saving PHCCs to the Syrian region apart from all aforementioned reasons. Despite the significance of the issue and the existing studies mentioned above, there is no study handling the PHCC location-allocation problem (identifying the right places to locate PHCCs so as to cover a maximum number of people suffering from health problems in the region with multi objectives) within the Syrian context. This implies that academic and scientific research interest in this field is still insufficient. These factors make this study a valuable contribution to literature. Therefore, due to the importance of the Syrian Crisis and the significance of its indirect global implications in recent years, this paper aims to bridge this gap and to be a valuable resource in such conflict areas by formulating a multi-objective model and integrating it into a real case study in the area. 

In this multi-objective model, as in [45], we have aimed to maximize the number of people served from the whole demand and minimize overall costs (as in [49]) at the same time. While accomplishing these objectives, we have focused on maximizing the number of located PHCCs with health availability factors (solar power, basement, internet service, laboratory service, blood grouping service and vaccination) and the cash for work amount of the located PHCCs simultaneously, differently from what was previously studied in the literature. These were accomplished through a mixed integer-weighted goal programming model developed for a real case study. The proposed model is solved by an optimization software. Another differentiating feature of this study is the use of real data directly collected from beneficiaries and consultants in the area.

As for the real case study, we selected Idleb Governorate, one of 14 governorates in Syria. There is an ongoing inflow of displaced people to the region and there are overcrowded camps and temporary shelters in the area. Livelihood and income are the most pressing needs in the governorate and these lead to difficulties in buying food and other basic items. Furthermore; schools, hospitals and other civilian infrastructure have been destroyed or damaged and access to basic services is deteriorating. Most of the PHCCs in Idleb are operated and funded by a local non-governmental organization or international non-governmental organization in coordination with the health directorate in the area. Although humanitarian non-governmental organizations are trying to relieve the suffering of people, they cannot fully respond to a long-term humanitarian crisis of such magnitude. These issues encouraged us to choose this governorate as the real case study for the purposes of identifying the locations of PHCCs and allocating people in the area to the located PHCCs so as to alleviate the impacts of this man-made disaster. 

Consequently, the remaining sections of this paper are organized as follows. In Section 2, the materials and methods employed in the study are described in detail. This section covers data collection and model formulation. The developed model is applied to solving the location-allocation PHCC problem in the area. Section 3 contains the results out of the relevant model and sensitivity analyses. Discussions and some suggestions for future studies are specified in Section 4. In the end, final remarks related to our work are indicated in Section 5. 

## 2. Materials and Methods 

### 2.1. Methodology

**Figure 1:**
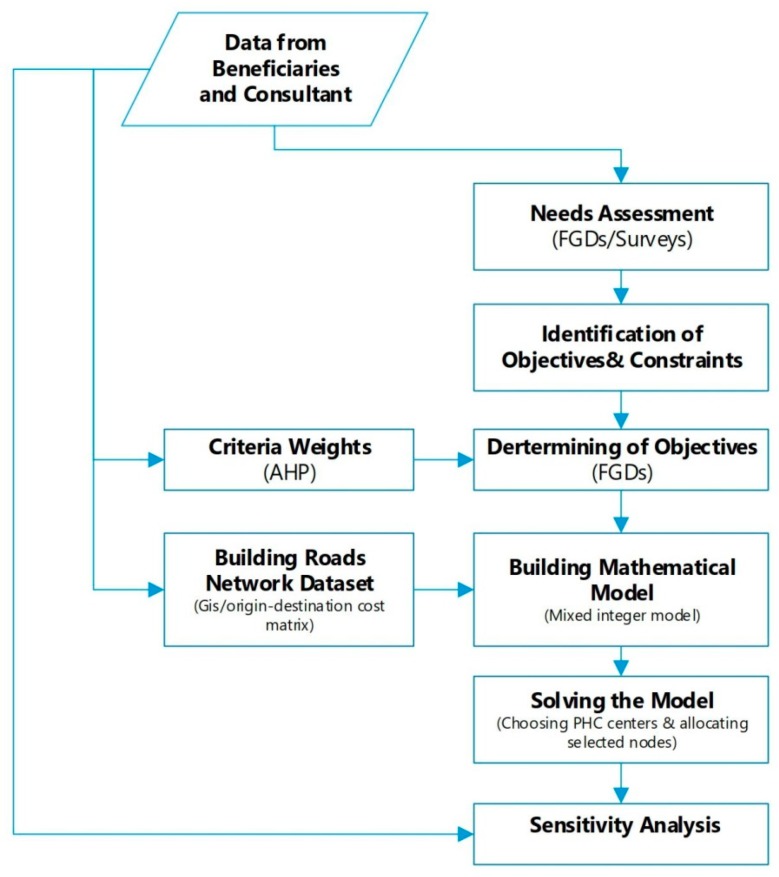
Proposed methodology adopted in the study.

Methodology adopted in this study for identifying PHCCs and allocating selected people to these PHCCs is demonstrated in Figure 1. Since direct beneficiaries are the most important stakeholders in this conflict area (and also in this study) and look forward to overcoming the detrimental impacts of the conflict, our approach is mainly based on direct beneficiaries. 

The methodology used contains four fundamental stages: the first stage of the methodology operated a needs assessment with the help of focus group discussions (FGDs), questionnaires, etc., in order to evaluate the needs of and the most significant issues for all relevant beneficiaries as the authorities required for publishing this data are not present in the conflict area. The data was collected between 5 March 2018 and 30 May 2018. Data regarding Idleb Governorate was extracted from a sample including 23 sub-districts within the governorate and a total of 338 communities by contacting direct beneficiaries and performing key source interviews with people who are aware of the issues in the society.

The second stage of the methodology was identifying the objectives and constraints of the model. This stage also involved the identification of priorities and penalties of objectives with regards to the beneficiaries and experts by weighting each objective by means of a multi-criteria decision making technique, Analytic Hierarchy Process (AHP). 

The third stage of the proposed methodology comprised of building the roads network dataset in the projected field utilizing the Geographic Information System (GIS) and updating it consistently by also covering risky roads with the aim of specifying origin-destination (OD) cost matrix by the distance between nodes (communities) and PHCCs. 

The fourth and last stage of the model was constructing the mathematical model with the introduction of a mixed integer model and solving it with the optimization package software so as to identify PHCCs and allocate people in the area to these centers. Moreover, sensitivity analyses were performed to discuss the results.

### 2.2. Building the Model

The problem addressed in this study was a part of the facility location-allocation problem, a branch of operations research associated with locating or positioning at least a new facility in between a number of existing or candidate facilities. The goal of this operations research specialty was optimizing (maximization/minimization) one or more objective functions such as profit, revenue, cost, travel distance, coverage, etc. Numerous fields of the application comprising private and public facilities, business areas, national and international fields are analyzed in the related literature [54]. Furthermore, facility location is studied by several researchers in terms of humanitarian relief [55,56,57,58,59]. 

It is possible to encounter multi-objective problems or multi-objective decision making (MODM) problems in the real world. By considering a myriad of interactions within the model, MODM methods endeavor to identify the best alternative which ideally satisfies the decision-maker (DM) by means of accomplishing satisfactory results for a set of objectives [54]. Yet, as in MODM, many real world decision making problems have contradictory objectives and this issue should be analyzed to obtain accurate results. In addition to this, an ideal solution in MODM is described as the one ending with an objective function’s optimum value at the same time within an effectual solution while none of the objective functions can be upgraded without damaging other objectives [60]. 

Thus, we took into account all these aspects and integrated facility location problems within MODM environment. Such problems might contain several objective functions to be achieved such as minimizing the total cost; maximizing the coverage; minimizing the longest distance from the existing facilities and maximizing the service, etc. 

In the light of this information, we introduced, through this paper, a mixed integer model incorporating capacitated maximal covering, fixed charge cost and some specific features of health centers in the Syrian context. These features are summarized as follows: 

Availability factors:

- Laboratory service,
- Blood grouping service,
- Vaccination,
- Solar power,
- Basement,
- Internet service.

Economic factors such as cash for work: Cash for work corresponds to the wages paid for workers to repair the PHCC and to make it habitable. 

Assumptions utilized in the model are stated below:

- Each demand node can be served as an entire unit from a PHCC or not served at all (0 or 1 without any fraction).
- Amount of each demand and its location are fixed.
- Paths throughout the updated built road networks are accessible and there is no broken or closed street.
- Variable costs for each allocated group of people at each location are related to the amount of people allocated in the PHCC irrespective of the PHCC’s location. It means that the cost of allocating a person to a certain PHCC is the same cost of allocating him/her to another PHCC in another location.

Consistent with the requirements for building the model, this paper uses the following parameters and variables for the multi-objective mixed integer model as shown in Table 1.

In the following parts, we demonstrate the multi-objective mixed integer model with objective functions and constraints.

Objective functions:

(1) $$Maximize\;\underset{i\in I}{\sum }\underset{j\in I}{\sum }a_{ij}\;Z_{ij}h_{i}$$

(2) $$Minimize\;\;\underset{j\in J}{\sum }f_{j}X_{j}+R\underset{i\in I}{\sum }\underset{j\in J}{\sum }\;Z_{ij}h_{i}+TC\underset{i\in I}{\sum }\underset{j\in J}{\sum }\;Z_{ij}h_{i}dis_{ij}$$

(3) $$Maximize\;\;\underset{j\in J}{\sum }SE_{j}X_{j}$$

(4) $$Maximize\;\;\underset{j\in J}{\sum }B_{j}X_{j}$$

(5) $$Maximize\;\;\underset{j\in J}{\sum }IS_{j}X_{j}\;\;$$

(6) $$Maximize\;\;\underset{j\in J}{\sum }Lab_{j}X_{j}\;\;$$

(7) $$Maximize\;\;\underset{j\in J}{\sum }BG_{j\;}X_{j}$$

(8) $$Maximize\;\;\underset{j\in J}{\sum }Vac_{j}X_{j}$$

(9) $$Maximize\;\;\underset{j\in J}{\sum }CW_{j\;}X_{j}$$

Subject to

(10) $$\sum_{j\in J}x_{j\;}\leq \;P$$

(11) $$\sum_{j\in J}z_{ij}\;\leq \;1\;\;\;\forall \;i\;\in \;I$$

(12) $$\sum_{j\in J}a_{ij}z_{ij}h_{i}\;\leq \;K_{j}\;\;\;\;\forall \;j\;\in \;J$$

(13) $$Z_{ij}\;\leq \;a_{ij}\;X_{j}\;\;\;\;\;\forall \;i\;\in \;I\;,\;\forall \;j\;\in \;J$$

(14) $$X_{j}\;,\;Z_{ij}\in \;[0,\;1].$$

Equations (1) to (9) correspond to the objective functions of the proposed multi-objective model. Equation (1) maximizes the number of people served out of the whole demand while Equation (2) minimizes the total cost covering three factors: a fixed cost which is the cost of setting up and opening a PHCC; a variable cost to run a PHCC within a year and a transportation cost to arrive at the related PHCC. Equations (3) to (8) maximize the number of located PHCCs with availability factors: Equation (3) maximizes the number of located PHCCs with the availability of solar power at location *j*; Equation (4) maximizes the number of located PHCCs with the availability of basement at location *j*; Equation (5) maximizes the number of located PHCCs with the availability of internet service at location *j*; Equation (6) maximizes the number of located PHCCs with the availability of laboratory service at location *j*; Equation (7) maximizes the number of located PHCCs with the availability of blood grouping service at location *j*; and Equation (8) maximizes the number of located PHCCs with the availability of vaccination at location *j*, respectively. Equation (9) maximizes the cash for work amount for located PHCCs. 

Constraints of the proposed model are presented in Equations (10) to (14). While Equation (10) limits the number of located PHCCs to be less than or equal to a specific value (*P*), Equation (11) guarantees that each demand can be covered mostly once. Equation (12) limits each PHCC to cover demand nodes with less capacity or a capacity equal to its own capacity. Equation (13) expresses that demand at node i∈I cannot be covered unless at least one of the PHCC sites covering node *i* is located. Binary variables for located PHCCs and covered nodes are presented in Equation (14).

Through an extensive literature review, we noticed that numerous methods have been built to deal with multi-objective problems [60,61,62,63,64,65,66,67,68]. Thus, the problem addressed in this paper is figured out via weighted goal programming. In the following section, we provide a brief introduction to weighted goal programming. 

### 2.3. Weighted Goal Programming (WGP)

Goal programming is one of numerous techniques for dealing with the modeling, solution, and analysis of multiple and conflicting objective problems. A traditional goal programming model contains constraints and a set of goals, all of which are taken into account simultaneously [69]. However, the final goal is to handle various objects which might be conflicting in the real world. This turns researcher’s attention to weighted goal programming (WGP), which is a type of goal programming and enables to optimize several objectives at once. This is achieved by converting crucial objectives (particularly those in contradiction) into goals and considering the remainder of objectives as constraints. Since trade-offs occur between objectives through deviation variables, negative and positive deviation variables are identified one by one for each goal corresponding to the over- and under-achievement of related goals. Hence, a single objective (achievement) function in the WGP minimizes the sum of undesirable deviations from the target goal values and results in a compromised solution between contradictory goals [70]. Any deviation is undesired, and the relative importance of each deviation variable is expressed by the relevant weights. They can be set either by expert estimation or a technique serving to that purpose (multi-criteria decision making techniques) [71]. 

Goals (namely a set of objectives) in WGP are commonly measured in different measurement units and they cannot be summed up as this would lead to incommensurability [72]. Deviations are scaled by utilizing the normalization technique to get rid of different units for various goals. Out of several normalization techniques (percentage normalization, Euclidean normalization, etc.); we employed percentage normalization in this paper. Thus, in relation to our objective functions with regard to cost (USD), allocated people (persons) and others, each deviation is converted into a percentage value apart from its target level. This enables to measure all deviations in the same units as a percentage. 

### 2.4. Analytic Hierarchy Process (AHP) for Identifying Criteria Weights in WGP

Our work contains multiple attributes and utilizes Multi Criteria Decision Making (MCDM) to address the relevant issues. MCDM techniques deal with decision making problems encompassing contradictory and miscellaneous criteria and objectives. At this point, it is noteworthy to point out that the Analytic Hierarchy Process (AHP) approach is very appropriate for group decision making as it contributes to numerous group preference collection methods [73,74]. AHP (developed by Saaty [75]) relies on expert judgments to obtain priority scales using pairwise comparisons. Throughout this process, comparisons are based on a scale of judgments (Table 2) that demonstrates to which extent one element dominates over another for a given attribute. Furthermore, it has theories to predict decision makers’ consistency of priorities [76]. Weights derived from the pairwise comparisons of AHP can be directly integrated into a WGP model [71]. Numerous studies reported the advantage of AHP for criteria weights [70,77,78,79,80,81,82,83]. Taking into account these features, it is employed in this study to weight objectives in WGP.

Table 2 demonstrates the rating scale utilized for pairwise comparisons in AHP. For detailed information about this method, readers should refer to [75].

In this paper, the pairwise comparison matrix of objectives was acquired by three expert decisions and their weights are presented in Table 3. 

The following equations (Equations (15) and (16)) were applied to check the consistency of responses acquired from decision experts. Equation (15) expresses the consistency index (CI) for a pairwise comparison matrix where $\lambda^{max}$ is the largest eigenvalue of the comparison matrix and *n* is the dimension of the matrix or the number of decision criteria. Equation (16) expresses the consistency ratio (CR) where RI(n) is a random index varying depending upon the size of matrix [76]. Random index values of random matrices are presented in Table 4.

(15) $$\mathrm{CI}=\;\frac{\lambda^{max}-n}{n-1}$$

(16) $$\mathrm{CR}=\;\frac{\mathrm{CI}}{\mathrm{RI}\left(n\right)}.$$

If the CR is equal to or less than 0.1, it is consistent and acceptable, however if it exceeds 0.1, the judgment sets may be too inconsistent to be reliable and the decision makers are asked to repeat pairwise comparisons to accomplish consistency in their responses. In our AHP, the CR was 0.005 meaning that it is consistent. 

### 2.5. Weighted Goal Programming Formulation

The weighted goal programming formulation of the problem is given below and then, modified equations are listed subsequently.

(17) $$Minimize\;\underset{n}{\sum }(p_{n}^{-}{}_{\;}d_{n}^{-}+p_{n}^{+}d_{n}^{+})\frac{1}{RHS_{n}}\;\left(\%\right).$$

Equation (17) minimizes the total deviations related to objective functions bearing in mind the penalty of each objective and percentage normalization according to $RHS_{n}$ “right hand sides” of the goal targeted for constraints from (18) to (26) as stated below. 

Thus, Equations (18) to (26) demonstrate the soft constraints taken into account in the weighted goal programming. 

Subject to:

(18) $$\sum_{i\in I}\sum_{j\in I}a_{ij}\;Z_{ij}h_{i}+d_{1}^{-}-\;d_{1}^{+}\;\;=\;RHS_{1}$$

(19) $$\sum_{j\in J}f_{j}X_{j}+R\sum_{i\in I}\sum_{j\in J}\;Z_{ij}h_{i}+TC\sum_{i\in I}\sum_{j\in J}\;Z_{ij}h_{i}\;dis_{ij}\;+d_{2}^{-}-\;d_{2}^{+}\;\;=\;RHS_{2}$$

(20) $$\sum_{j\in J}SE_{j}X_{j}+d_{3}^{-}-\;d_{3}^{+}\;\;=\;RHS_{3}$$

(21) $$\sum_{j\in J}B_{j}X_{j}+d_{4}^{-}-\;d_{4}^{+}\;\;=\;RHS_{4}$$

(22) $$\sum_{j\in J}IS_{j}X_{j}\;+d_{5}^{-}-\;d_{5}^{+\;}=\;RHS_{5}$$

(23) $$\sum_{j\in J}Lab_{j}X_{j}+d_{6}^{-}-\;d_{6}^{+}\;=\;RHS_{6}$$

(24) $$\sum_{j\in J}BG_{j}X_{j}\;+d_{7}^{-}-\;d_{7}^{+}\;=\;RHS_{7}$$

(25) $$\sum_{j\in J}Vac_{j}X_{j}+d_{8}^{-}-\;d_{8}^{+}\;=\;RHS_{8}$$

(26) $$\sum_{j\in J}CW_{j}X_{j}+d_{9}^{-}-\;d_{9}^{+}\;=\;RHS_{9}.$$

In the weighted goal programming model, Equations (10) to (14) are utilized in the same way as described before.

## 3. Case Study and Results 

### 3.1. Case Study

By way of utilizing the methodology detailed in Section 2, we have performed a case study in Idleb Governorate of Syria. Idleb Governorate is situated in the northwest of Syria, has a border with Turkey and it has been a conflict area since the Syrian crisis started in 2011. It has an approximate area of 6097 km^2^ and the population estimate of the Governorate for 2010 (prior to the war) was about 1,464,000. Due to the crisis, no updated population estimate is available for the area. Furthermore, because of the crisis in the country, there are fluctuations in population and many people have immigrated from Aleppo, Eastern Ghouta, Homs or Daraa to Idleb. It might have evolved in this way because Idleb seems to be safer when compared to other Governorates in Syria and this makes it a good place for the settlement of internally displaced people (IDPs). Hence, our data collection in the area resulted in a population of 1,852,440 people. 

**Figure 2:**
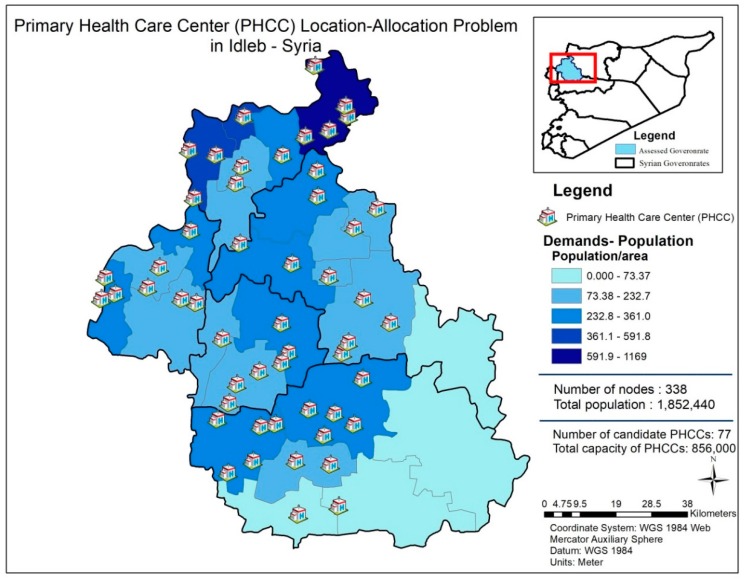
Distribution of nodes and candidate Primary Health Care Centers (PHCCs) for the relevant location-allocation problem.

Figure 2 demonstrates the distribution of nodes and candidate PHCC locations for the location-allocation problem addressed in the study. 

Data regarding the following parameters of the proposed model was collected by operating an assessment via surveys and FGDs:

- Demands at nodes;
- Availability factors of candidate PHCCs (solar power, basement, internet service, laboratory service, blood grouping service and vaccination);
- Coverage distance;
- Fixed cost of locating a PHCC at candidate locations;
- Capacity of each candidate location;
- Cash for work amount at each candidate location.

More information about the parameters described above in the form of charts and figures is available in this interactive link (https://goo.gl/x2GjVv). 

**Figure 3:**
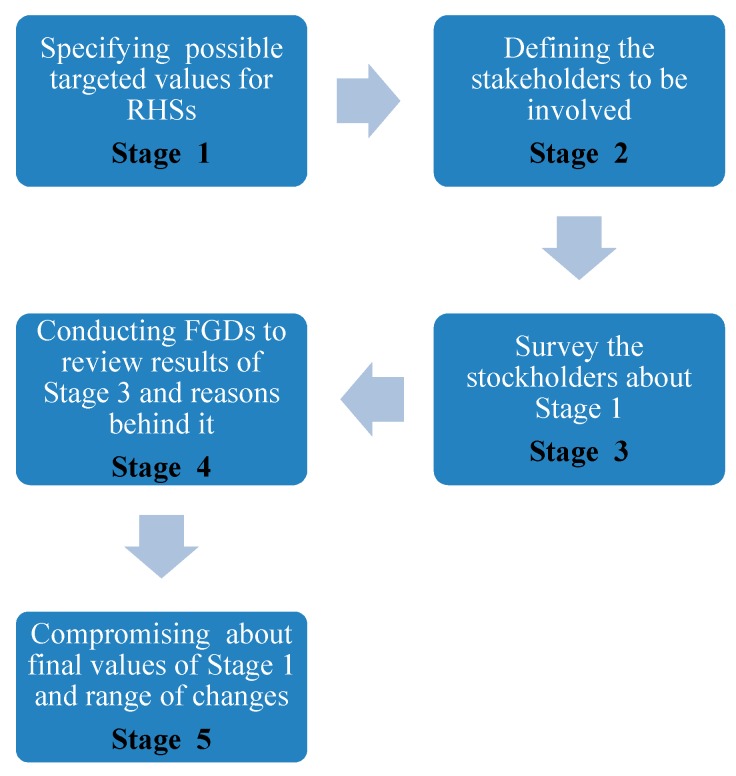
Flowchart of focus group discussions (FGDs)/surveys utilized in the study.

The study, as displayed in Figure 2, covers 338 nodes and identifies 77 candidate PHCCs with the aforementioned fixed costs, variable costs and availability factors. Of the 77 candidate PHC centers, the maximum number of located/selected PHCCs are designated as 60 (*p* = 60), due to budgetary and management considerations, since the maximum number of possible PHCCs is 77 and if beneficiaries and experts are determined to identify a larger number, it means that they need more procedures and regulations to control it. On the contrary, if they consider a smaller number to be selected, it means that they will not be able to allocate a lot of people in this vulnerable area. As a result, they have found a compromise throughout FGDs/surveys (please see Figure 3 for detailed information about the flowchart of this process). Distances between nodes and candidate locations are generated utilizing GIS (ArcMap 10.4.1, Esri, Redlands, CA, USA). In this process, a roads network dataset is built by constructing the Origin-Destination (OD) matrix. 

The desired $RHS_{n}$, $p_{n}^{-}$ and $p_{n}^{+}$ values which will be utilized in the weighted goal programming model are acquired according to the process in Figure 3 and AHP are as follows:

- Regarding the RHS of our first objective, the target value of allocated people, we have set our target value as 1,852,440 following data collection since we aim to allocate all people in the case study area.
- Among candidate PHCCs; 31 have laboratory service, 33 have blood grouping service, 60 have vaccination, 18 have solar power, 36 have basement and 65 have internet service. Through the process in Figure 3, we determined these objective’s target values as: 30, 30, 30, 18, 30 and 30, respectively.
- According to the results obtained by AHP and depicted with Table 3; for every objective function(*n*); $p_{n}^{-}$ values are set as: $p_{1}^{-}$ 45, $p_{2}^{-}$ 0, $p_{3\;}^{-}$ 5, $p_{4}^{-}$ 5, $p_{5}^{-}\;$5, $p_{6}^{-}\;$5, $p_{7}^{-}$ 5, $p_{8}^{-}$ 9 and $p_{9}^{-}\;$9. $p_{n}^{+}$ values are set as: $p_{1}^{+}$ 0, $p_{2}^{+}$ 14, $p_{3}^{+}$ 0, $p_{4}^{+}$ 0, $p_{5}^{+}\;$0, $p_{6}^{+}\;$0, $p_{7}^{+}$ 0, $p_{8}^{+}$ 0 and $p_{9}^{+}\;$ 0. Here, goals though 1 to 9 correspond to: allocated people objective, total cost objective, cash for work, solar power, basement, internet service, laboratory service, blood grouping service and vaccination, respectively.
- Total cost budget and cash for work target values are determined as USD 1,000,000 and USD 100,000 via the process in Figure 3.

Then, demands, candidate locations and constraints are taken into account and the problem is solved via an optimization package software. 

The process of identifying the aforementioned values via FGDs with key stakeholders is composed of five stages and its flowchart is demonstrated in Figure 3 below. 

In the first stage, possible targeted values of RHSs to be considered depending on similar projects and situations are identified. The second stage defines main stakeholders that are connected with the specific value (financial aspect, humanitarian context, administration as well as donors, partners, beneficiaries, representatives, etc.) and will be involved in the next stages and achieves the related value. In the third stage, stakeholders are surveyed to find out their opinions and recommendations supported by reasons and clarifications. The fourth stage involves conducting FGDs to examine the results acquired in the third stage and discussing them. In the fifth and last stage of this process, estimated values are compromised by establishing the highest consensus value for most of the stakeholders by considering each value’s range of changes to be handled in sensitivity analyses until achieving a specific point “allocating as many people as possible” or “the full capacity of PHCCs”. 

### 3.2. Results

**Figure 4:**
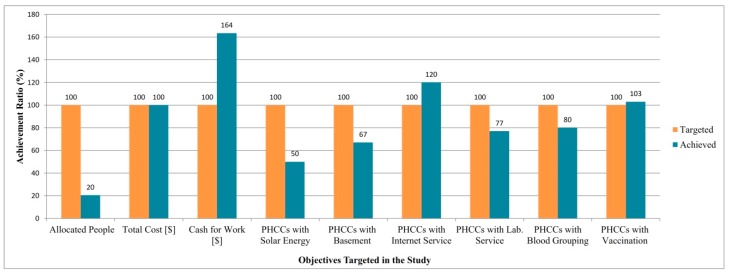
Outputs achieved in the study as a percentage of the targeted level.

We solved the model and attained the results demonstrated in Figure 4. It depicts achievement ratios (%) compared to the objectives targeted in the study. 

Since we aim to achieve every objective by 100% (horizontal orange column in Figure 4), it can be observed that four objectives acquire this (blue columns in Figure 4). For instance, we fulfilled an achievement ratio of 100% in terms of total cost budget. We fulfilled an achievement ratio of 120% and 103% for the objectives of PHCCs with internet service and PHCCs with vaccination, respectively. These mean that the number of located PHCCs with these availability factors is higher than the targeted values. Due to the conditions in Syria and Idleb, this is a significant and positive situation. Hence, the higher the number of PHCCs fulfilling these availability factors is, the higher the number of people benefitting from these centers is. We observe that achievement ratios for the objectives “PHCCs with laboratory service” and “PHCCs with blood grouping” are below the achievement ratio of 100% (77% and 80%, respectively). Achievement ratios for PHCCs with solar power and PHCCs with basement are 50% and 67%, respectively. A good compromise was achieved regarding these four results. The value of the objective “cash for work” is momentous for humanitarian contexts such as Syria-Idleb because the cash for work provided to vulnerable families as wages in return for working can alleviate the suffering of such persons in conflict areas. It is seen that this objective is achieved by 164% in this study, which is a crucial positive feature of the results achieved out of the model. 

Most importantly, we realized that our goal of “allocated people” did not achieve the targeted level (20%) because the total capacity of candidate PHCCs is 856,000. Since it is a conflict area and people are seeking health care, our model aimed to allocate all people (1,852,440 people) in the relevant area of Idleb. We aimed to include a maximum of 60 PHCCS out of 77 available candidates and the model located 42 PHCCs in total. 

**Figure 5:**
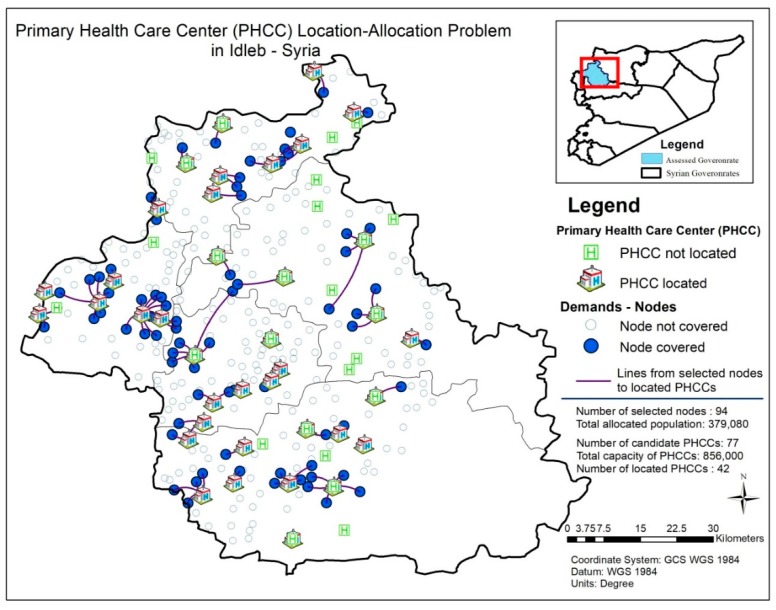
Results of the PHCC location-allocation problem addressed in the study.

Moreover, the number of allocated people (and all other objectives) depends mostly on the cost budget and it includes multiple factors: fixed cost, running cost and transportation cost. Within a cost budget of USD 1,000,000, the model allocates 379,080 people, which corresponds to an achievement ratio of 20% approximately. Since all our objectives except the number of allocated people, PHCCs with solar power, PHCCs with basement, PHCCs with laboratory service and PHCCs with blood grouping service achieved the targeted level, we focused on improving these objective values after general analyses. Figure 5 highlights the results of the PHCC location-allocation problem addressed in the study by demonstrating the nodes covered, the PHCCs located and the covered nodes allocated to the located PHCCs all together on a map. It also contains the summary of the addressed problem. The model selects 94 nodes out of 338 nodes by allocating them to the located 42 PHCCs. The total allocated population is 379,080 people and this corresponds to 44% of the PHCC’s total capacity, which is 856,000.

**Figure 6:**
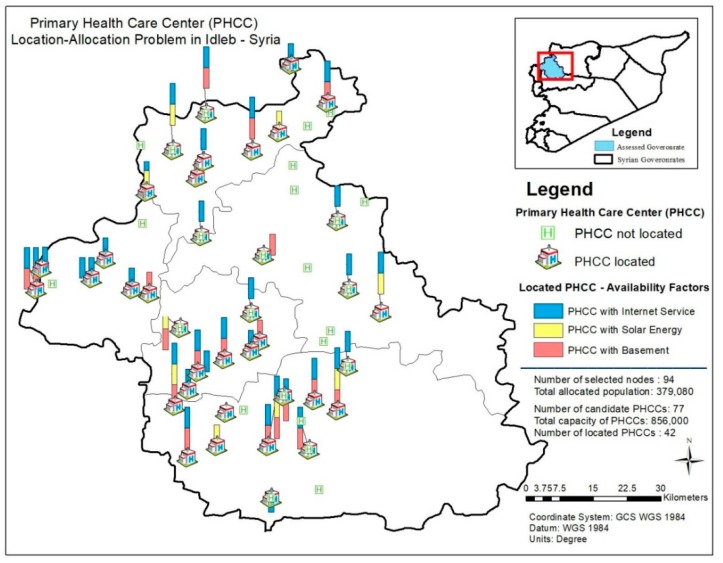
Availability of internet service, solar power and basement for the located PHCCs.

**Figure 7:**
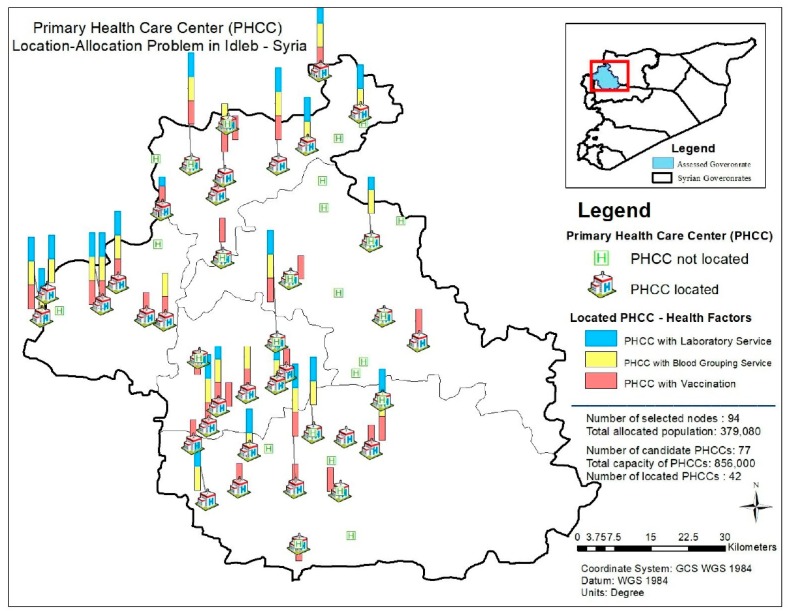
Availability of health factors for the located PHCCs.

Figure 6 and Figure 7 demonstrate the availability of internet service, solar power and basement of the located PHCCs and the availability of health factors (laboratory service, blood grouping service and vaccination) regarding the results of the PHCC location-allocation problem. In Figure 6, PHCCs with the availability of internet service are symbolized by blue bars; with the availability of solar power by yellow bars and with the availability of basement by orange bars. In Figure 7, blue bars display the located PHCCs with laboratory service while yellow bars and orange bars display the ones with blood grouping service and vaccination, respectively.

### 3.3. Sensitivity Analysis

**Figure 8:**
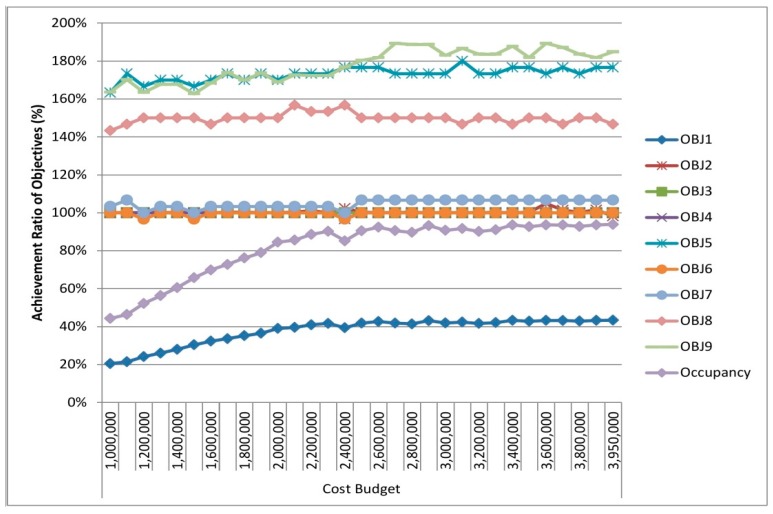
Achievement ratios of objectives acquired as a result of conducting sensitivity analyses regarding the cost budget.

**Figure 9:**
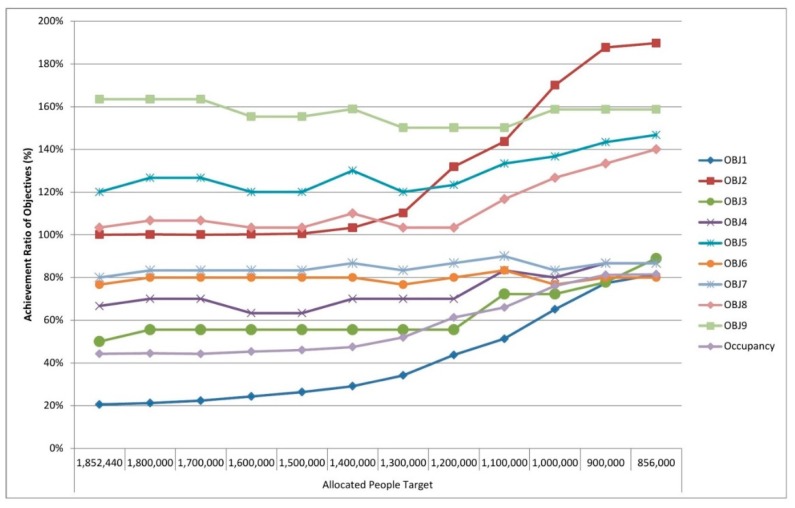
Achievement ratios for objectives as a result of conducting sensitivity analyses in the target of allocated people.

**Figure 10:**
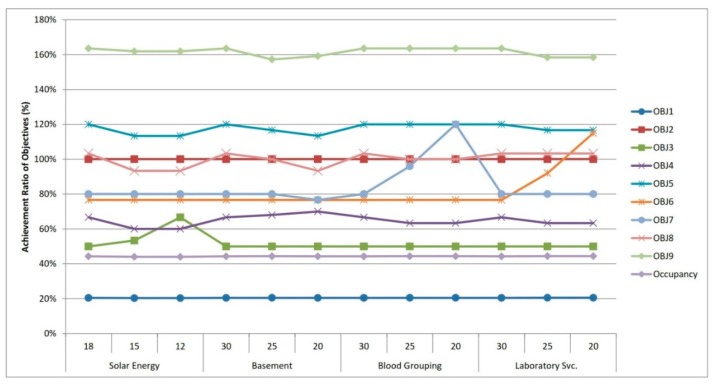
Achievement ratios for objectives as a result of conducting sensitivity analyses regarding availability factors.

In this part, we dealt with sensitivity analyses from three perspectives: (i) according to the model results, some objectives are under the targeted achievement ratio. However, it is a conflict area and our primary target is allocating as many people as possible, thus our main goal is to improve the objective of “coverage.” Since coverage, the number of allocated people, depends mainly on the cost budget within the study, a sensitivity analysis was implemented by increasing the cost budget within a specific range while keeping other inputs constant to find out how sensitive the objective regarding the number of allocated people is to these changes. This analysis enables us to observe other objectives’ changes as the cost budget is increased. (ii) We conducted sensitivity analyses through which the target of allocated people is decreased within a specific range until the total capacity of PHCCs is utilized in order to observe the achievement ratio of all objectives. (iii) As for the availability factors, the achievement ratio of which is under the targeted ratio (solar power, basement, laboratory service and blood grouping service), we decreased the RHS values of these parameters within a specific range so as to detect changes regarding other objectives. The following Figure 8, Figure 9 and Figure 10 display the results of the aforementioned sensitivity analysis. 

In these figures, OBJ1 to OBJ9 correspond to the following descriptions, respectively:

OBJ1    : Value achieved in the Coverage; 

OBJ2    : Value achieved in the Total Cost;

OBJ3    : Value achieved in the located PHCCs with Solar Power; 

OBJ4    : Value achieved in the located PHCCs with Basement;

OBJ5    : Value achieved in the located PHCCs with Internet Service;

OBJ6    : Value achieved in the located PHCCs with Laboratory Service;

OBJ7    : Value achieved in the located PHCCs with Blood Grouping Service;

OBJ8    : Value achieved in the located PHCCs with Vaccination;

OBJ9    : Value achieved in the Cash for work,

Occupancy : Occupancy ratio of PHCCs (obtained by dividing the total number of allocated people to the total capacity of PHCCs).

Figure 8 demonstrates the graph of achievement ratios for objectives acquired as a result of conducting sensitivity analysis by changing the cost budget. In this analysis, we increased the total cost budget by USD 200,000 at every model run, and observed the results regarding objectives 1 to 9. Although there is no significant difference on the achievements rates from USD 2,600,000 to USD 3,950,000, we achieved the maximum number of allocated people at USD 3,950,000. Through this cost budget, the model allocated 803,180 people to the 60 located PHCCs of which 18 have solar power, 30 have basement, 53 have internet service, 30 have laboratory service, 32 have blood grouping service and 44 have vaccination. Within this cost budget, the value of cash for work is acquired as USD 184,852. While the model firstly allocated 379,080 people, which corresponds to 20% of total PHCCs capacity occupancy, the model allocated 803,180 people by obtaining a PHCC occupancy rate of 94% after sensitivity analysis. All related achievement ratios are available in Figure 8. There are two reasons for the failure to acquire an achievement ratio of 100% regarding the occupancy parameter: firstly, since we located PHCCs and there is a coverage distance defined as the maximum distance people can travel to reach to the PHCC, some people could not be allocated to any located PHCC even if all PHCCs are located. Secondly, even if a PHCC is located, in the event that the number of people in the demand node nearby is higher than that PHCC’s remaining capacity, this node cannot be covered by that PHCC and the remaining capacity of PHCC will be the same without serving to any more people. 

The population of the case study is 1,852,440 but in addition to this, the total capacity of candidate PHCCs is 856,000. By considering that it is a conflict area and all people in the field will require health care, we targeted to allocate all of the population in the first place. However, since PHCCs currently have a specific capacity, we decreased the targeted number of allocated people within a specific range to observe results and achievement ratios in this part of the sensitivity analysis. Figure 9 demonstrates the achievement ratios of objectives as a result of conducting a sensitivity analysis within the target of allocated people. If we aim to allocate 856,000 people by keeping all other parameters in the model constant, the model allocates 697,500 people to the located 52 PHCCs. Even though we aim to utilize the PHCCs in full capacity and occupancy, the model can at most allocate 81% of the targeted allocated people to 81% of PHCCs’ total capacity mostly due to a limited cost budget of USD 1,000,000 in these analyses. Achievements ratios for other objectives can be obtained from Figure 9. 

With regard to the last sensitivity analysis, we focused on four availability factors: solar power, basement, blood grouping service and laboratory service. We decreased the target values (RHS values) of these availability factors and the results of achievement ratios for all objectives are depicted in Figure 10. This figure indicates that changing the RHS values of these availability factors almost does not affect the achievement ratios of other objectives. These changes only impact the achievement ratios of these objectives. In Figure 10, these changes can be tracked by following the green line with square markers for solar power (OBJ3); the purple line for basement (OBJ4); the orange line for laboratory service (OBJ6) and the light blue line for blood grouping service (OBJ7).

## 4. Discussion

In this study, we employed a multi-objective decision-making methodology to identify the optimum PHCC locations for the people in the north of Syria-Idleb, and to allocate the selected people nodes to the located PHCCs. Since it is necessary to consult with direct beneficiaries in humanitarian context as they are suffering from a myriad of miseries and we are trying to relieve their miseries, we mostly counted on direct beneficiaries because of the absence of authorities that can directly provide such data. 

In the first stage of our four-stage method, we operated a needs assessment with the help of focus group discussions, questionnaires, etc., so as to assess the needs of people. In the second stage, we set the objectives and constraints of the proposed model throughout AHP and FGDs. We constructed the roads network dataset utilizing the Geographic Information System and updated it in association with risky roads in order to build the origin-destination cost matrix in the third stage. In the last stage of the model, we built the mixed integer model, adapted it into a weighted goal programming model and solved it via an optimization package software and conducted a set of sensitivity analyses. 

After the execution of the model, it allocated 379,080 people with a cost of USD 1,000,353 and a cash for work of USD 163,549. A total of 42 PHCCs were located by considering the constraints and objectives identified in advance based on the region’s humanitarian context, the needs of people in the area and the indicators of stakeholders. Of these 42 PHCCs, nine PHCCs have solar power, 20 PHCCs have basement, 36 PHCCs have internet service, 23 PHCCs have laboratory service, 24 PHCCs have blood grouping service and 31 PHCCs have vaccination.

To improve the results achieved in the analysis, we conducted sensitivity analyses and encountered three cases: (i) since the monetary budget is limited, the model managed to allocate 20% of people. Thus, the monetary budget should be increased to achieve better results in locating more PHCCs and allocating more people to them. (ii) As the main focus of this study is to allocate people to PHCCs and they have capacity constraints, the capacity of PHCCs should be increased in order to allocate and serve more people in this vulnerable area. (iii) Although some objectives are under the targeted level of achievement, changing their RHS values hardly affects the achievement ratios of other objectives. This action only affects their own achievement ratios.

This paper focuses only on the northern part of Syria and assesses the relevant PHCCs in terms of nine criteria. Our primary aim was to identify the locations of PHCCs within Idleb Governorate and to relieve the miseries of the people in the area. Thus, labor factors and medical resources are not included in our model. These are the limitations of this paper and researchers can consider the following suggestions within their future studies so as to overcome these deficiencies:

- PHCCs or health care facilities can be assessed with more criteria such as the availability of running water and availability of electricity in hours.
- Criteria such as education, access to food and water can also be handled alongside the criteria/objectives addressed in this study.
- In future studies, labor factors (doctors, nurses, technicians and guards) and medical resources (beds, drugs, etc.) can be included in the model.
- Sensitivity analyses can be performed by changing multiple parameters simultaneously.
- Other regions of Syria can also be added into the relevant area, which can make the paper more comprehensive.
- A web-based tool can be designed incorporating the mathematical model and GIS and adapted to various similar problems.
- A dynamic model might be proposed to deal with the high degree of uncertainty regarding such problems.
- The problem can be handled by different techniques such as heuristic, meta-heuristic methods, hybrid models and social simulations.
- A conflict risk assessment can be applied to investigate the connection between the risk of armed conflict/ongoing crisis and a set of indicators such as education, infrastructure and access to health care facilities and food.

## 5. Conclusions

A conflict can influence human life in every aspect, particularly if it takes place in a densely-populated area and lasts for almost 8 years, as in Syria and specifically in Idleb. In this humanitarian emergency environment, people live in refugee camps, seek asylum and they even need basic items such as food, water, blanket, etc., apart from health. People need health care in every environment, but this proves to be more significant especially in a conflict area because conditions are more desperate since they live in harsh conditions, they are prone to infectious diseases, and they cannot access health care, which brings about an elevated mortality rate. The literature review revealed that, although there are numerous studies about primary health care subjects in natural and man-made disasters, there are no studies related to the location-allocation problem regarding PHCCs. Accordingly, there is no study handling the PHCC location-allocation problem within the Syria context which means that academic and scientific interest to this field is still unsatisfactory. These were the motives of this paper and therefore, this paper focused on identifying the locations of PHCCs and allocating people in the area to the located PHCCs with the aims of bridging this gap and being a valuable resource in such conflict areas. These contributions are attained by formulating a multi-objective model and integrating it into a real case study in the area so as to alleviate the dire impacts of this man-made disaster. 

From a humanitarian perspective, the proposed methodology is applicable in conflict areas to achieve the most feasible solution by combining the multi-objective mathematical model with GIS by utilizing real data from the area. These findings are expected to enable benefactors to respond to the needs of people, especially in these humanitarian contexts. 

Last but not the least, this paper’s main objective is to ensure accessibility to this model by any country, authority or institution. The purpose of this paper is to be useful for all humanitarian contexts and other services. In this paper, we identified the optimum location of PHCCs under specific constraints in a conflict area. Even decision variables, parameters and criteria may differentiate depending on the specific framework of the related problem area (this problem area can be general service centers, education, or other services), but our model can be used for different situations in other countries or conflict areas. 

## Figures and Tables

**Table 1:** Meanings of model parameters and variables.

Parameter	Meaning
*I*	Set of demand nodes; *i* *ϵ* *I*
*J*	Set of candidate locations; *j ϵ J*
*N*	Set of targeted goals; *n ϵ N*
$h_{i}$	Demand at node *i*
$f_{j}$	Fixed cost of locating a PHCC at site *j*
R	Running cost of each person at site *j* (constant number)
$K_{j}$	Capacity of each candidate location *j*
$CW_{j}$	Amount of cash for work in each candidate location *j*
TC	Transportation cost for a distance of 1 km (constant number)
$dis_{ij}$	Distance between node *i* and location *j* (acquired via constructing a GIS roads network dataset)
$a_{ij}$	$\{\begin{array}{c} 1\\ 0 \end{array}\begin{array}{l} \;\;\;\;if\;demand\;at\;node\;i\;can\;be\;covered\;by\;PHCC\;center\;at\;j\\ \;\;\;\;otherwise \end{array}\;$ (covering matrix)
$Lab_{j}$	$\{\begin{array}{c} 1\\ 0 \end{array}\begin{array}{l} \;\;if\;PPHCC\;at\;location\;j\;has\;laboratory\;service\;\\ \;\;otherwise \end{array}$
$BG_{j}$	$\{\begin{array}{c} 1\\ 0 \end{array}\begin{array}{l} \;\;if\;PPHCC\;at\;location\;j\;has\;blood\;grouping\;service\;\;\;\\ \;\;otherwise \end{array}$
$Vac_{j}$	$\{\begin{array}{c} 1\\ 0 \end{array}\begin{array}{l} \;\;if\;PHCC\;at\;location\;j\;has\;vaccination\;\\ \;\;otherwise \end{array}$
$SE_{j}$	$\{\begin{array}{c} 1\\ 0 \end{array}\begin{array}{l} \;\;if\;PHCC\;at\;location\;j\;has\;solar\;energy\;\;\;\\ \;\;otherwise \end{array}$
$B_{j}$	$\{\begin{array}{c} 1\\ 0 \end{array}\begin{array}{l} \;\;if\;PHCC\;at\;location\;j\;has\;basement\;\;\;\\ \;\;otherwise \end{array}$
$IS_{j}$	$\{\begin{array}{c} 1\\ 0 \end{array}\begin{array}{l} \;\;if\;PHCC\;at\;location\;j\;has\;internet\;service\;\;\;\\ \;\;otherwise \end{array}$
$p_{n}^{-}$	Penalty of not achieving the objective related to deviation $d_{n}^{-}$
$p_{n}^{+}$	Penalty of not achieving the objective related to deviation $d_{n}^{+}$
$RHS_{n}$	Right hand sides of targeted goal *n* according to goal programming
$X_{j}$	$\{\begin{array}{c} 1\\ 0 \end{array}\begin{array}{l} \;\;\;\;if\;we\;locate\;a\;PHCC\;at\;candidate\;location\;j\\ \;\;\;\;otherwise \end{array}$
$Z_{ij}$	$\{\begin{array}{c} 1\\ 0 \end{array}\begin{array}{l} \;\;\;\;if\;demand\;at\;node\;i\;is\;served\;by\;a\;PHCC\;at\;location\;j\\ \;\;\;\;otherwise \end{array}$
$d_{n}^{+}$	Positive deviational variable—amount of an overachieved targeted goal *n*
$d_{n}^{-}$	Negative deviational variable—amount of an underachieved targeted goal *n*

**Table 2:** Rating scale utilized in Analytic Hierarchy Process (AHP) (adopted from Saaty [75]).

Intensity of Importance	Definition	Explanation
1	Equal importance	Two elements are equally important
3	Moderate importance	Experience and judgment slightly favor one element over another
5	Strong importance	Experience and judgment strongly favor one element over another
7	Very strong importance	One element is favored very strongly over another
9	Extreme importance	One element is absolutely more important over another
2, 4 ,6, 8	Intermediate values	When compromise is needed

**Table 3:** Pairwise comparisons with the final weight of each objective by AHP.

Objectives	Weights
P1	44.7%
P2	14.4%
P3	4.7%
P4	4.7%
P5	4.7%
P6	4.7%
P7	4.7%
P8	8.7%
P9	8.7%

**Table 4:** Random index values [76].

N	3	4	5	6	7	8	9	10	11
RI(n)	0.58	0.9	1.12	1.24	1.32	1.41	1.45	1.49	1.51
